# FANTOM enters 20th year: expansion of transcriptomic atlases and functional annotation of non-coding RNAs

**DOI:** 10.1093/nar/gkaa1054

**Published:** 2020-11-19

**Authors:** Imad Abugessaisa, Jordan A Ramilowski, Marina Lizio, Jesicca Severin, Akira Hasegawa, Jayson Harshbarger, Atsushi Kondo, Shuhei Noguchi, Chi Wai Yip, Jasmine Li Ching Ooi, Michihira Tagami, Fumi Hori, Saumya Agrawal, Chung Chau Hon, Melissa Cardon, Shuya Ikeda, Hiromasa Ono, Hidemasa Bono, Masaki Kato, Kosuke Hashimoto, Alessandro Bonetti, Masaki Kato, Norio Kobayashi, Jay Shin, Michiel de Hoon, Yoshihide Hayashizaki, Piero Carninci, Hideya Kawaji, Takeya Kasukawa

**Affiliations:** RIKEN Center for Integrative Medical Sciences, Yokohama, Kanagawa, Japan; RIKEN Center for Integrative Medical Sciences, Yokohama, Kanagawa, Japan; Advanced Medical Research Center, Yokohama City University, Kanagawa, Japan; RIKEN Center for Integrative Medical Sciences, Yokohama, Kanagawa, Japan; RIKEN Center for Integrative Medical Sciences, Yokohama, Kanagawa, Japan; RIKEN Center for Integrative Medical Sciences, Yokohama, Kanagawa, Japan; RIKEN Center for Integrative Medical Sciences, Yokohama, Kanagawa, Japan; RIKEN Center for Integrative Medical Sciences, Yokohama, Kanagawa, Japan; RIKEN Center for Integrative Medical Sciences, Yokohama, Kanagawa, Japan; RIKEN Center for Integrative Medical Sciences, Yokohama, Kanagawa, Japan; RIKEN Center for Integrative Medical Sciences, Yokohama, Kanagawa, Japan; RIKEN Center for Integrative Medical Sciences, Yokohama, Kanagawa, Japan; RIKEN Center for Integrative Medical Sciences, Yokohama, Kanagawa, Japan; RIKEN Center for Integrative Medical Sciences, Yokohama, Kanagawa, Japan; RIKEN Center for Integrative Medical Sciences, Yokohama, Kanagawa, Japan; RIKEN Center for Integrative Medical Sciences, Yokohama, Kanagawa, Japan; Database Center for Life Science, Research Organization of Information and Systems, Mishima, Shizuoka, Japan; Database Center for Life Science, Research Organization of Information and Systems, Mishima, Shizuoka, Japan; Database Center for Life Science, Research Organization of Information and Systems, Mishima, Shizuoka, Japan; Program of Biomedical Science, Graduate School of Integrated Sciences for Life, Hiroshima University; RIKEN Center for Integrative Medical Sciences, Yokohama, Kanagawa, Japan; RIKEN Center for Integrative Medical Sciences, Yokohama, Kanagawa, Japan; RIKEN Center for Integrative Medical Sciences, Yokohama, Kanagawa, Japan; Karolinska Institutet, Stockholm, Sweden; RIKEN Head Office for Information Systems and Cybersecurity, Wako, Saitama, Japan; RIKEN Head Office for Information Systems and Cybersecurity, Wako, Saitama, Japan; RIKEN Center for Integrative Medical Sciences, Yokohama, Kanagawa, Japan; RIKEN Center for Integrative Medical Sciences, Yokohama, Kanagawa, Japan; RIKEN Center for Integrative Medical Sciences, Yokohama, Kanagawa, Japan; RIKEN Center for Integrative Medical Sciences, Yokohama, Kanagawa, Japan; RIKEN Center for Integrative Medical Sciences, Yokohama, Kanagawa, Japan; RIKEN Preventive Medicine & Diagnosis Innovation Program, Wako, Saitama, Japan; Research Center for Genome & Medical Sciences, Tokyo Metropolitan Institute of Medical Science, Tokyo, Japan; RIKEN Center for Integrative Medical Sciences, Yokohama, Kanagawa, Japan; Institute for Protein Research, Osaka University, Suita, Osaka, Japan

## Abstract

The Functional ANnoTation Of the Mammalian genome (FANTOM) Consortium has continued to provide extensive resources in the pursuit of understanding the transcriptome, and transcriptional regulation, of mammalian genomes for the last 20 years. To share these resources with the research community, the FANTOM web-interfaces and databases are being regularly updated, enhanced and expanded with new data types. In recent years, the FANTOM Consortium's efforts have been mainly focused on creating new non-coding RNA datasets and resources. The existing FANTOM5 human and mouse miRNA atlas was supplemented with rat, dog, and chicken datasets. The sixth (latest) edition of the FANTOM project was launched to assess the function of human long non-coding RNAs (lncRNAs). From its creation until 2020, FANTOM6 has contributed to the research community a large dataset generated from the knock-down of 285 lncRNAs in human dermal fibroblasts; this is followed with extensive expression profiling and cellular phenotyping. Other updates to the FANTOM resource includes the reprocessing of the miRNA and promoter atlases of human, mouse and chicken with the latest reference genome assemblies. To facilitate the use and accessibility of all above resources we further enhanced FANTOM data viewers and web interfaces. The updated FANTOM web resource is publicly available at https://fantom.gsc.riken.jp/.

## INTRODUCTION

This year marks the 20th year from the first publication by the FANTOM Consortium, where biology, genomics and bioinformatics experts from all over the world collaborated in providing the first collection and functional annotation of more than one million full-length mouse cDNAs (1). During the 20 years following the initial publication, the FANTOM consortium have not only contributed to, but also evolved genomics research. This was achieved by developing and applying novel technologies to create big datasets in each subsequent edition of the project (2). In the fifth edition of FANTOM (FANTOM5), we constructed ‘atlases’ of promoters (3,4), enhancers (5), miRNAs (6) and long non-coding RNAs (lncRNAs) (7) in several mammalian genomes. Recently, the sixth edition of FANTOM (FANTOM6) was launched to elucidate the biological functions of lncRNAs (8). LncRNAs constitute a major fraction of the transcriptome and can be broadly categorized into sense-intronic, antisense, intergenic, divergent and enhancer RNA regions (2). Although some of the lncRNAs characteristics such as their rapid evolution were already known (9,10), conservation of lncRNA sequences and genomic position are suggestive of functional potential (11–13). Currently, only a relatively small number of lncRNAs have any experimentally confirmed functions, compared to the tens of thousands of lncRNAs reported in the literature and available in databases such as Ensemble (14) or GENCODE (15). In the pilot phase of FANTOM6, we used a high-throughput screening (HTS) platform to systematically knockdown 285 lncRNAs in human dermal fibroblast cells and profiled the molecular phenotypes by cap analysis of gene expression (CAGE) (16) protocol; followed then by downstream bioinformatics analyses.

**Figure 1.**
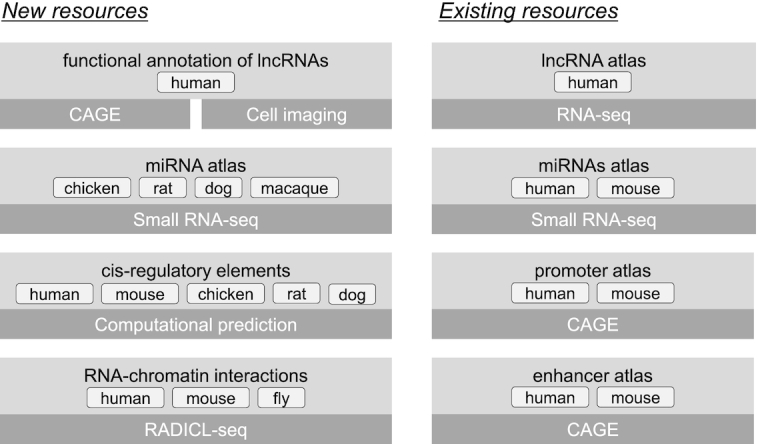
Overview of FANTOM5 and FANTOM6 web resources: new and existing resources generated in FANTOM5 and FANTOM6 projects. The light-gray boxes show the atlas or dataset and the dark-gray boxes show the method used to generate them.

Here, we report the latest updates of the FANTOM web resource. Figure 1 shows the summary of the FANTOM web resource from FANTOM5 and FANTOM6 projects including previously reported and currently updated resources (17,18), as well as newly-released datasets (8,16,17). Mainly, we provide new non-coding RNA (ncRNA) resources including the functional annotation of 285 human lncRNAs from the FANTOM6 pilot study (8), along with the miRNA atlases of rat, dog, chicken and macaque (19). Each miRNA atlas was also supplemented with computationally-predicted motif activities, genome-wide transcription factor binding sites (TFBS) and multiple genomic alignments of human, mouse, rat, chicken and macaque. Another newly-added resource, is a dataset generated using RADICL-seq, a recently published method to identify genome-wide RNA-chromatin interactions (20), which can potentially make contributions to inferring the functions of lncRNAs. Moreover, the FANTOM5 sample metadata was further curated and improved upon to include additional ontology terms. In parallel, the existing miRNA and CAGE datasets for several species (human, mouse and chicken) were remapped to their latest genome assemblies. Finally, we enhanced the web resources and upgraded user interfaces for better performance and usability.

## RESULTS

### Functional annotation of 285 lncRNAs in human fibroblast cells

**Figure 2.**
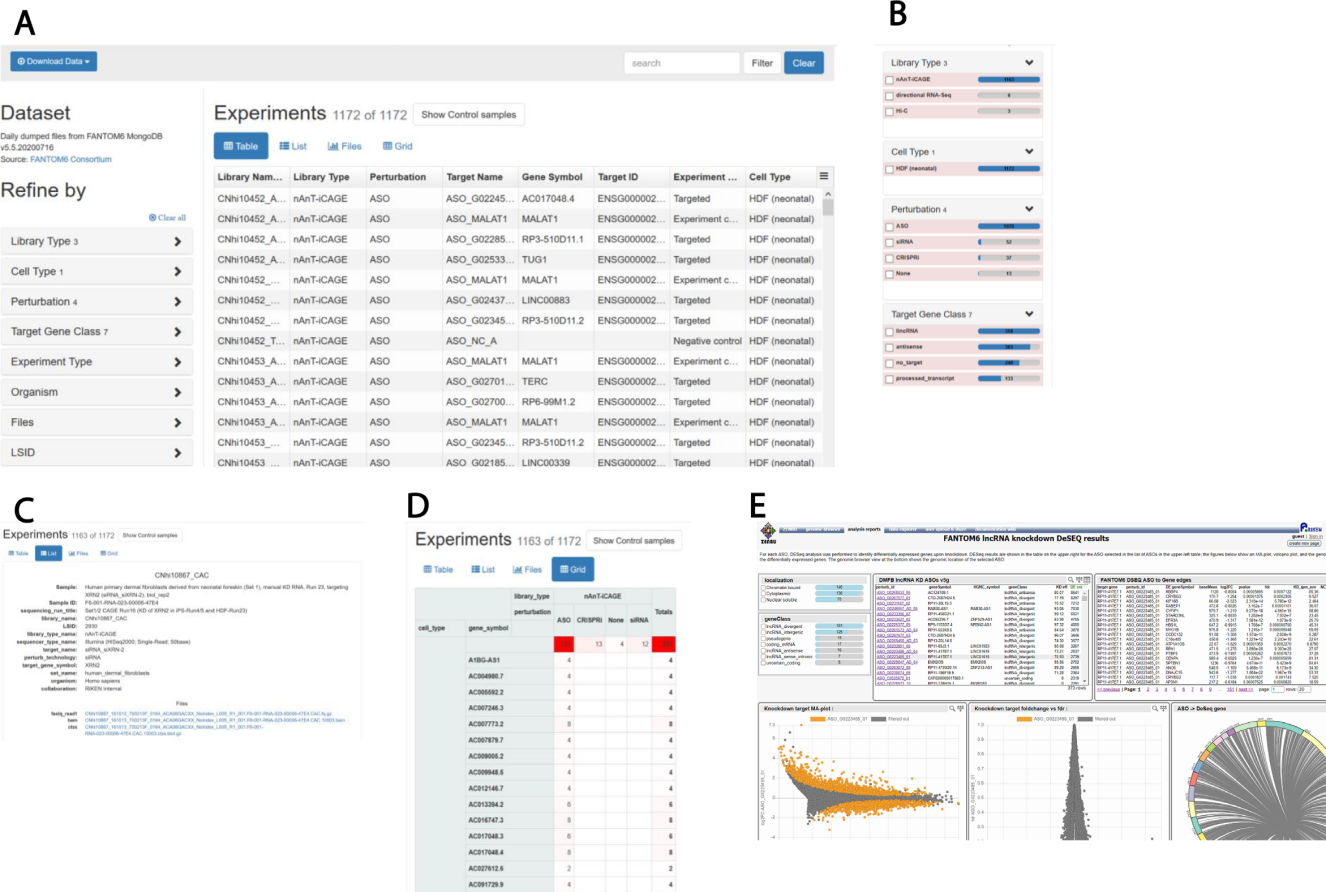
Access to the FANTOM6 data web resources: (**A**) web resources interface for FANTOM6 experiments in tabular view for each CAGE library with associated attributes; (**B**) web resource experiments view can be refined by the check-box and adjusted; (**C** and **D**) provide a list, files and a grid view, respectively, of selected experiments as in (B); (**E**) analysis results of FANTOM6 pilot project using ZENBU analysis report view.

The major goal of FANTOM6 is to systematically elucidate the function of lncRNAs in the human genome. For this, we used LNA antisense GapmeR technology to suppress the expression levels of a set of known and novel lncRNAs (7) in different cell types. In the pilot project (8) we selectively targeted 285 lncRNAs in human fibroblast cells using 5–15 non-overlapping antisense oligos (ASOs) in an automated high-throughput cell culture platform. We then evaluated the cellular phenotypic changes using Incucyte^®^ live imaging and found that over 25% of the targeted lncRNAs affected cell growth and morphology, as well as cell migration (8). For the subset of 154 lncRNAs with high knock-down efficiency (generally >50%), we used deep-CAGE sequencing and profiled transcriptomes of 970 knock-down and control libraries. We then assessed the functional implications of the 340 targeted individual ASOs by performing differential gene expression, Motif Activity Response Analysis (MARA) (21), and Gene Set Enrichment Analysis (GSEA) (22). The resulting molecular phenotype recapitulated the observed cellular phenotypes while providing additional insights into the affected genes and pathways. In summary, we can now disseminate the largest (to-date) lncRNA knockdown dataset with molecular phenotyping (Figure 2A–D); this can be further explored using our interactive portal (Figure 2E). The interfaces and dataset download can be found at https://fantom.gsc.riken.jp/6/.

### miRNAs atlas of chicken, rat, dog and macaque

**Figure3.**
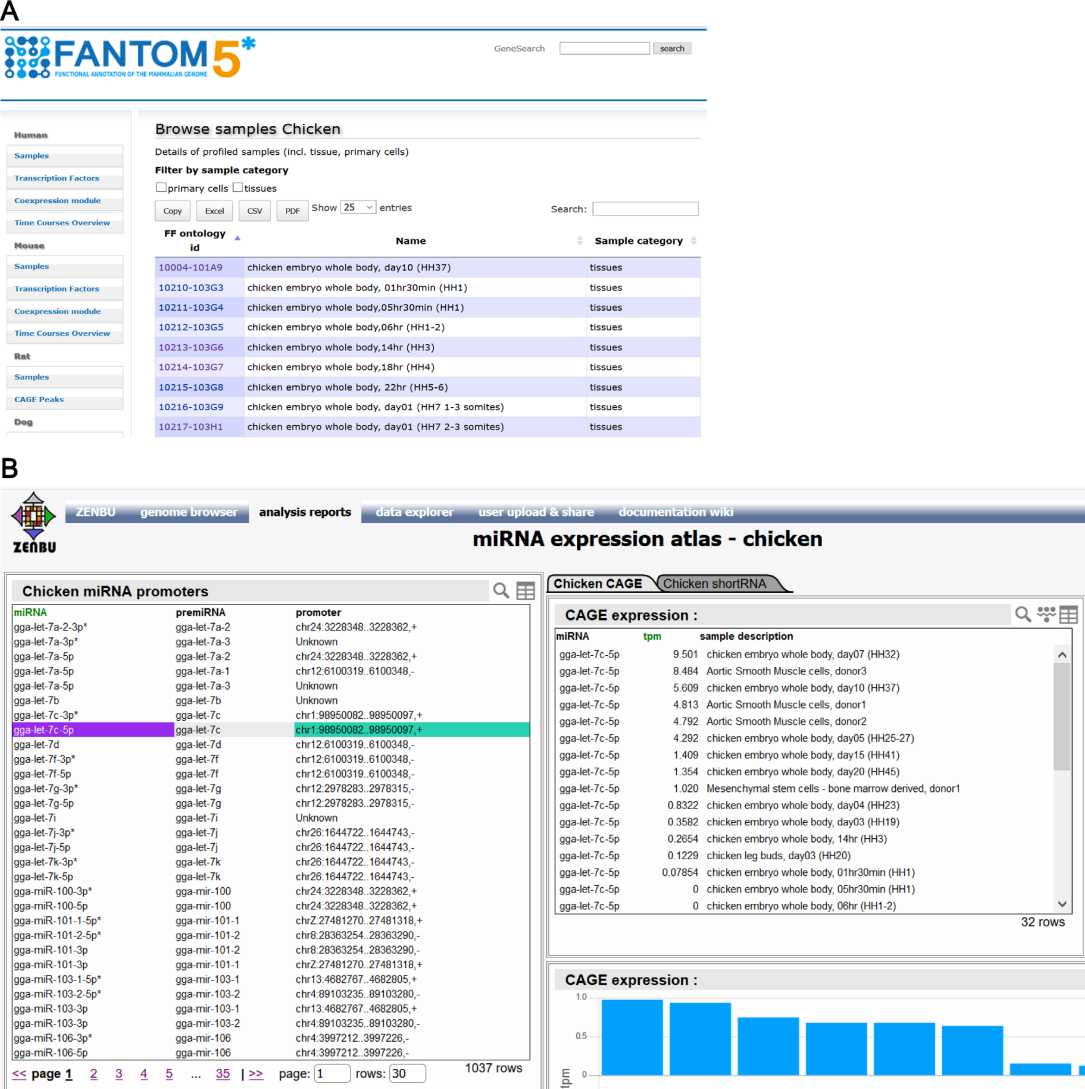
Access to the miRNA atlas sample metadata and analysis results: (**A**) the list of chicken, rat and dog samples and their metadata are provided in FANTOM5 SSTAR resource; (**B**) ZENBU report for miRNA atlas allows for interactive browsing of miRNAs detected in given samples and for exploring their promoter(s) expression. The raw reads and alignments are available through the FANTOM5 data files repository.

miRNAs are a class of small non-coding RNAs that regulate mRNAs, post-transcriptionally, by either inhibiting their translation or by inducing their degradation (23). To profile the expression of miRNAs in different cell types, FANTOM5 generated short RNA (sRNA) sequencing data for 386 human and 42 murine RNA samples, most of which were obtained from primary cells (6). Additional sRNA sequencing data, produced in rat, dog and chicken for aortic smooth muscle primary cells; this allowed for an evolutionary analysis of miRNA expression patterns in this cell type (19). The data, together with FANTOM5 sRNA sequencing data of universal RNA samples, were further used to predict candidate novel miRNAs in these three species (19). All sequencing data mapped to genome assemblies rn6 (rat), canFam3 (dog) and galGal5 (chicken) are available as BAM files from https://fantom.gsc.riken.jp/5/data/. The FANTOM5 miRNA expression atlas can be accessed through a newly created visualization system at https://fantom.gsc.riken.jp/zenbu/reports/#FANTOM_miRNA_atlas. In addition to the expression profiles and associated annotations of each miRNA, the atlas also provides the putative promoter of the corresponding primary miRNA transcript, as identified by analysing FANTOM5 CAGE data, in each of five species (Figure 3). A list of samples used in this atlas and their associated metadata was made available via SSTAR (Semantic catalog of Samples, Transcription initiation And Regulators), https://fantom.gsc.riken.jp/5/sstar/ a semantic database of FANTOM5 samples (24).

### Putative promoters in rat, dog and chicken

In the FANTOM5 project, promoters were revealed by identifying peaks in transcription initiation signals captured by CAGE. For quantitative analyses, a strict (*’robust’*) threshold was applied to expression levels of actively transcribed regions, whereas a more relaxed (‘*permissive’*) threshold was used for qualitative analysis aimed at covering lowly expressed regions. Here, we release new sets of CAGE peaks for rat, dog, and chicken, based on the permissive threshold and following the same approach and parameters as in our previous study (4). These new data sets were utilized for identification of enhancers and miRNA promoters (16) in the three species, and made available for further analysis at https://zenodo.org/record/3856413.

### Multiple genome alignments, genome-wide TFBS predictions and motif activity analysis

Cis-regulatory elements, in the promoter region of each gene, are a key contributor to the variation in gene expression observed between different cell types (4) and species (19). We used MotEvo (25) to generate genome-wide predictions of transcription factor binding sites (TFBSs), using position-weight matrix models obtained from SwissRegulon (26). The evolutionary model used by MotEvo requires a multiple genome alignment, which was available from existing sources for human and mouse, but was generated *ab initio* for rat, dog, and chicken, each of these against 29 vertebrate species (19). These multiple genome alignments, as well as the genome-wide TFBS predictions, for human (genome assembly hg19 and hg38), mouse (mm9 and mm10), rat (rn6), dog (canFam3) and chicken (galGal5 and galGal6) (19) are available at https://fantom.gsc.riken.jp/5/suppl/Alam_et_al_2020/. The genome-wide TFBS predictions can be used in a wide variety of bioinformatics analyses, including motif activity analysis; a methodology to infer the regulatory activity of transcription factors (TFs) by decomposing CAGE expression levels of promoters based on the presence of TF binding sites in each promoter (21). Scripts to perform motif activity analysis, using the provided genome-wide TFBS predictions, are available at https://fantom.gsc.riken.jp/5/suppl/Alam_et_al_2020/.

### Human, mouse and Drosophila dataset for genome-wide RNA-chromatin interactions by RADICL-seq

FANTOM projects identified a large number of lncRNAs in the mammalian genome (7,11). Many of these RNAs are found in the cell nucleus and are bound to the chromatin. However, it is not yet understood which RNAs and what DNA genomic regions interact specifically with each other in different cell types. To better understand these interactions, we developed a new technology called RNA And DNA Interacting Complexes Ligated and sequenced (RADICL-seq) (17), which maps genome-wide RNA-chromatin interactions in intact nuclei. For the development of the technology, we generated and sequenced mouse ES cell and OPC (oligodendrocyte progenitor cells) libraries. Additionally, RADICL-seq sequencing data were produced in human iPSC and Drosophila S2 DRSC cells as a pilot experiment to expand the project and to ultimately uncover RNA-chromatin interactomes in multiple cell types. All sequencing data, mapped to genome assemblies hg38 (human), mm10 (mouse), and dm6 (Drosophila) are available as BEDPE files from https://fantom.gsc.riken.jp/6/datafiles/RADICL-Seq/.

### Reprocessing of the miRNA atlases with the latest reference genome assemblies

The original FANTOM5 human and mouse miRNA atlases (6) were based on the GRCh37 assembly for human and the GRCm37 for mouse. Just as we processed our human and mouse promoter atlases (27), we remapped all human and mouse short RNA-seq libraries to the latest GRCh38 and GRCm38 reference genome assemblies; provided by the Genome Reference Consortium (https://www.ncbi.nlm.nih.gov/grc). The CAGE and short RNA-seq alignments for chicken, which were previously based on the galGal5 reference genome, were remapped to the latest galGal6 genome assembly. All reprocessed datasets are available in our *‘reprocessed’* section at (https://fantom.gsc.riken.jp/5/datafiles/reprocessed/).

### Expansion of the ontology annotations in sample metadata

We revised the metadata for FANTOM5 human and mouse samples to better facilitate the usage of the corresponding datasets. The update includes additional annotations of cancer cell line samples based on NCI Thesaurus (https://ncit.nci.nih.gov/), as well as refined annotations of cell and tissue samples based on Cell Ontology (28) and UBERON (29) respectively. These updates also improve the interconnectivity to the RefEx database (https://refex.dbcls.jp/) (30). The revised metadata files are available on our web site at (https://fantom.gsc.riken.jp/5/datafiles/reprocessed/metadata_latest/).

### Updates of viewers and interfaces

To serve the research community via FANTOM web portals and databases (31), the FANTOM web-based interfaces are continually updated to provide reliable and user-friendly resources. In the latest update, the metadata for the rat, dog, chicken and macaque libraries used in a report by Alam, T *et al.* (16) were added to the FANTOM5 SSTAR at https://fantom.gsc.riken.jp/5/sstar/. ZENBU genome browser, an interactive tool for visualization and analysis of large-scale sequencing datasets (32), provides a genomic view of several FANTOM atlases; it was updated to accommodate recently published data for macaque, see http://fantom.gsc.riken.jp/zenbu/gLyphs/#config=TJD69JeXM5ylAJTRKiJ45D.

FANTOM5 utilized the UCSC Genome Browser data hub (33) and setup the FANTOM5 data hub, which provided the TSS activities in individual biological states, and the corresponding identified regions in human and mouse genomes https://fantom.gsc.riken.jp/5/datahub/description.html. Recently, the FANTOM5 data hub was updated by adding individual tracks for chicken, dog, rat and macaque using aforementioned dataset updates, as described in (16).

The FANTOM5 metadata are exported to the RIKEN MetaDatabase (https://metadb.riken.jp/) (34). Here, the metadata for RIKEN’s life-science databases are integrated and openly provided to researchers world-wide with the semantic web technology. The FANTOM5 metadata in the RIKEN MetaDatabase is available at https://metadb.riken.jp/metadb/db/fantom5.

### Conclusion and future updates

The latest updates of the FANTOM web resource consist mainly of the enhancement and expansion of ncRNA resources. These updates cover the latest published functional annotations of 285 human lncRNAs; the miRNAs atlas of rat, dog, chicken and macaque; and preliminary results of genome-wide RNA–chromatin interaction with the new RADICAL-seq protocol; and aim to contribute to uncovering the functions of ncRNAs. Moreover, the FANTOM web resource has also been updated by (i) adding computationally predicted resources of motif activity; genome-wide TFBS and multiple genome alignments for human, mouse, chicken, rat and macaque; (ii) reprocessing of the existing atlases and datasets based on the latest-available genome assemblies; (iii) further curating metadata for FANTOM5 sample ontologies; and (iv) enhancing existing web applications and user interfaces. These updates will help researchers to better understand transcriptomes of these profiled species, facilitate cross-species studies and improve our understanding of the biological functions of mammalian ncRNAs. The contribution of the FANTOM consortium to genomics research will further continue by providing additional datasets and resources to enhance our understanding of mammalian genomes and the function of lncRNAs, including those from the ongoing FANTOM6 projects.
